# A Study of the Flexural Properties of PA12/Clay Nanocomposites

**DOI:** 10.3390/polym14030434

**Published:** 2022-01-21

**Authors:** Josip Stojšić, Pero Raos, Andrijana Milinović, Darko Damjanović

**Affiliations:** Mechanical Engineering Faculty in Slavonski Brod, University of Slavonski Brod, 35000 Slavonski Brod, Croatia; praos@unisb.hr (P.R.); amilinovic@unisb.hr (A.M.); ddamjanovic@unisb.hr (D.D.)

**Keywords:** nanocomposites, polyamide 12, clay, mechanical properties, mixing

## Abstract

Polymer nanocomposites consist of a polymer matrix and reinforcing particles that have at least one dimension under 100 nm. The processing of nanocomposite polymers is the most important stage, determining the final properties of nanocomposites. Nanocomposites are now preferentially prepared by melt-mixing using conventional compounding processes such as twin-screw extrusion. Many processing parameters (polymer matrix type, content and type of nanofiller, barrel temperature, screw speed, number and shape of extruder screws, etc.) affect the properties of nanocomposites. This research work represents an investigation of the influence of processing parameters (amount of nanoclay filler, the screw rotation speed, and extruder barrel temperature) on the flexural properties of polyamide 12/nanoclay-reinforced nanocomposite. From the test results, it is apparent that an increase in nanoclay content from 1 to 8% significantly increases flexural strength. The obtained nanocomposite has a 19% higher flexural strength and a 56% higher flexural modulus than pure PA12. Mathematical models that show the dependence of flexural strength and flexural modulus on the processing parameters used were obtained as a result of this analysis.

## 1. Introduction

Composite materials, which have played an important role in many fields for a long time, are mostly used in the automotive, aviation, and space industries; shipbuilding; electrical and industrial systems; orthopedic parts; and the construction industry. Generally, polymer nanocomposites are composites consisting of a polymer matrix containing a dispersion of nanoscale particles. Nanocomposites can be prepared in solution or by in situ polymerization, but today nanocomposites are preferentially prepared by melt-mixing using conventional compounding processes such as twin-screw extrusion [1,2,3,4,5].

The performance of nanocomposites depends on the properties on their constituents; on their composition; and on various characteristics of the nanoparticles, such as their size, aspect ratio, specific surface area, and physical/chemical compatibility with the matrix. Due to the large surface area of nanosize particles, only small amounts are needed to cause significant changes in the mechanical (Young’s modulus and strength), physical, thermal, and electrical properties of polymer nanocomposites. In this way, preferably better properties of nanocomposites (compared with conventional microcomposites) can be achieved [4,6].

Layered silicates (so-called clays) are the most studied class of nanoscale fillers because they can improve many material properties. They can be added to existing materials at a relatively low cost. Studies have shown that nanocomposites that consist of a polymer and layered silicate have significantly improved properties when compared to neat polymer or conventional composites at both macro- and micro-scales [6,7].

Montmorillonite clay is one of the most used sheet silicate materials in polymer nanocomposites. A single clay platelet has a thickness of about 1 nm. However, clay platelets tend to stack together into larger micron-sized aggregates that are electrostatically held together. The use of only a small percentage of montmorillonite, with the fully dispersed layers in the polymer matrix, will lead to a much higher interfacial area of the polymer and the filler compared with conventional microcomposites [2].

The extent of clay dispersion will vary depending on the interaction between the polymer and the clay surface, as well as the thermomechanical stresses applied during the melt mixing. The process starts with the diffusion of polymer chains within the clay interlayer spacing (intercalation stage), followed by the delamination of the individual platelets (exfoliation stage) and their diffusion into the melt. There are three different polymer–clay morphologies that may result from melt mixing. Microcomposites are obtained when the polymer is unable to diffuse into the interlayer spacing and the clay remains in its agglomerate state, creating a micro-dispersed phase. An intercalated nanocomposite exhibits a multilayer morphology due to the diffusion of polymer chains into the interlayers, whose spacing is approximately 2–4 nm. An exfoliated morphology consists of individual clay platelets suspended in a polymer melt (with the distance between them exceeding 8–10 nm) [2,8]. An exfoliated structure is preferred for a polymer composite because it produces the largest matrix–filler contact area, which leads to the best nanocomposite properties [2,8,9].

The mechanical properties of composites have been studied extensively, mostly through experiments but also through additional computational methods [10,11]. Modelling and optimization techniques used allow researchers to find the best combination of constituent materials and processing parameters for obtaining optimal properties of the resulting composites [12,13,14]. Several statistic-based techniques are used for the optimization of processing variables; one of these is the response surface method (RSM). This method investigates the relationship between input and output variables and allows the mathematical modelling of the system [15]. The RSM mostly relies on the statistical regression method, as it is practical, economical, and relatively easy to use [16].

Choi at al. used RSM to optimize the polymerization conditions in a thermoplastic-resin transfer molding process for CFPA6 composite. The obtained regression model has been described to be appropriate for estimating the tensile strength of CFPA6 composite materials dependent on the injection speed, activator ratio, and catalyst ratio [17].

Pragasam at al. used the Box–Behnken response surface design for the investigation of the flexural strength of cellulose microfibrils-reinforced composite and to obtain optimized parameter results [18].

Samuel at al. used the Taguchi approach and general regression analysis for the optimization and modeling of the flexural strength of the PxGyEz composite. The obtained mathematical models describe the flexural behavior of the developed composite with a good correlation with the experimental values [14].

To understand the influence of the fabrication process parameters on the mechanical properties of composites and optimize them, Athijayamani at al. used ANOVA and developed a regression equation for predicting the tensile and flexural strength of nano-hybrid wood polymer composites [19]. Other authors also used statistical tools for their investigations [20,21,22,23]. 

According to the available literature and experiments carried out by other authors, it was concluded that mechanical properties of nanocomposites have a good correlation with the type of clay used and the clay dispersion. Additionally, clay dispersion correlates with the process parameters, the type of extruder used, and the screw configuration. All of these play important roles in achieving a good organoclay dispersion and excellent mechanical properties. Additionally, from these papers it cannot be concluded how to set the mixing parameters to obtain the highest value of the mechanical properties or, more precisely, flexural properties. 

In all cases, the best exfoliation will be achieved when the structure of the surfactant and the process parameters are optimized [24,25].

In previous works, the influence of the mixing parameters used on polyamide nanocomposites has been investigated, but only on tensile or thermal properties and with different reinforced particles (forming natural particles and fibers into carbon nanotubes) [6,26,27,28,29].

In none of these papers have the mixing parameters and content of Cloisite 93 A in a polyamide 12 matrix been investigated. Hocine and Follain investigated the influence of mixing parameters of PA12 matrix nanocomposites reinforced with Cloisite 30B on the tensile and thermal properties [6,29,30].

Since this combination of material matrix and filler (PA12 and cloisite 93A) has not been sufficiently investigated, in this research the influence of mixing parameters on the structure and flexural properties of PA12/clay nanocomposites was investigated in order to find the optimal combination of constituent materials and processing parameters for obtaining the optimal flexural properties of PA12 composites reinforced with Cloisite 93A nanoclay.

## 2. Materials and Methods

### 2.1. Materials

The material used for the matrix is PA12 made by Eos GmbH (Krailling, Germany), with the trade name EOSINT P PA2200, charge nr. 919613. Due to its excellent properties (i.e., high strength, good chemical and UV resistance, high resolution, biocompatibility) and low cost, PA12 is widely used for the production of laser-sintered parts (prototypes as well as end-use parts). PA2200 is a white polyamide 12 powder with an average grain size of 60 μm and a bulk density of 0.445 g/cm^3^ according to DIN53466 0.435- [31].

The material used for the reinforcement was Cloisite 93A nanofiller made by Southern Clay Products (part of BYK Additives and Instruments, Wesel, Germany). Cloisite 93A is an additive used in plastics for improving various plastic physical properties, such as reinforcement, HDT, and barrier properties. This material is a modified natural MMT made using a ternary ammonium salt with a concentration of 90 meq/100 g. According to the manufacturer, the d-spacing (001) is 2.36 nm, which results in a diffraction angle (2*θ*) of 3.7° [32].

### 2.2. Design of Experiment

A number of manufacturing parameters have an influence on the final properties of nanocomposites. In this study, the influence of nanoclay content, screw rotation frequency, and mixing temperature on the flexural properties of PA12/clay nanocomposite was investigated. Independent variables with high and low values were:

A: Nanoclay content (from 3 to 9%) as factor 1;

B: Screw rotation frequency (from 20 to 40 min-1) as factor 2;

C: Mixing temperature (from 210 to 230 °C) as factor 3.

For this study, a central composite design of experiment with axial points out of the plane was selected. There are five levels of factors (coded by -1.682; -1; 0; 1; 1.682) and 19 experiments: 2^3^ factorial points, 2 × 3 axial points, and 5 center points (Table 1).

### 2.3. Preparation of Specimens

Mixtures with different nanoclay contents were made and each mixture was extruded using parameters selected according to the design of the experiment. Extrusion was performed on the (Brabender GmbH & Co. KG, Duisburg, Germany) extrusion line equipped with a twin-screw extruder, cooling paths, and a granulator. Technical data of the extrusion line are given in Table 2.

Due to small L/D ratio, each mixture was extruded three times because longer residence times in the extruder favor better the dispersion of nanoclay [24]. 

After extrusion, polymer strands were granulated and afterwards formed into 125 × 125 × 2 mm plates using direct molding at a melting temperature of 215 °C and a molding pressure of 15 MPa. It is also known that the parameters of the molding process have an impact on the properties of the polyamide composite-molded parts. All plates and nanocomposites were made from pure PA12 using the same conditions to make the impact the same for every plate [36]. 

The shape and dimensions of the test specimens used for the determination of the flexural properties are specified in HRN EN ISO 178:2019, paragraph 6.1.3. “Other test specimens” [37]. Test specimens with dimensions of (*l* × *b* × *h*) 40 × 25 × 2 mm were cut from the obtained plates. 

In order to avoid fractures caused by low toughness, plates were preheated at 80 °C during the cutting of specimens. For each experiment, 5 specimens were made (95 specimens in total), and, in addition, 5 specimens were made out of pure PA12.

### 2.4. Morphological and Structural Characterization

Morphological and structural characterization was carried out using XRD and SEM analyses. X-ray diffraction patterns were recorded on the X,pert PRO diffractometer (Malvern Panalytical Ltd, Malvern, United Kingdom) using CuKα radiation with a wavelength of 1.54 A°. An acceleration voltage of 40 kV and filament current of 30 mA were applied. The samples were scanned at a rate of 0.05°/min from 1° to 30° of 2*θ*. 

A SEM analysis of polyamide 12/ clay nanocomposites was performed on a VEGA/TESCAN LMU (TESCAN ORSAY HOLDING, a.s., Brno – Kohoutovice, Czech Republic) with an operating voltage of 10.0 KV and a magnification of 5 KX.

### 2.5. Determination of Flexural Properties of PA12/Clay Nanocomposites

**Figure 1:**
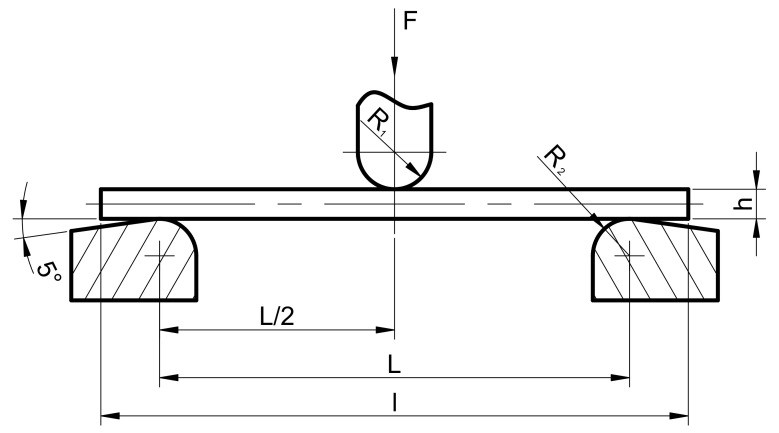
Position of test specimen at the start of the test [37].

The determination of the flexural strength and flexural modulus was conducted in accordance with the HRN EN ISO 178:2019. This document specifies a method for determining the flexural properties of plastic using test specimens that can be molded or machined from finished or semi-finished products. The preferred specimen dimensions are also specified within this document. The test specimen, supported by a beam, was deflected at a constant rate at the midspan until it fractured or until the deformation reached a predetermined value. During this procedure, the force applied to the test specimen was measured. Two supports and a central loading edge were arranged as shown in Figure 1 [37].

According to the HRN EN ISO 178:2019, *R*_1_ = 5 mm ± 0.1 mm and *R*_2_ = 2 mm ± 0.2 mm were used for a test specimen with a thickness ≤ 3 mm, *h* = 2 mm, width of *b* = 25 mm, and length of *l =* 40 mm [37].

According to the HRN EN ISO 178:2019, the flexural strength *σ*_fM_ is the maximum flexural stress that can be sustained by the test specimen during a bending test. This can be calculated using the equation:

(1) $$\sigma_{\mathrm{fM}}=\frac{3F_{M}\cdot L}{2b\cdot h^{2}}$$

where *σ*_fM_ (MPa) is the maximum flexural stress, *F*_M_ (N) is the maximum applied force, *L* (mm) is the span between supports (32 mm), *b* (mm) is the width of the specimen, and *h* (mm) is the thickness of the specimen.

Flexural modulus *E*_f_ is the ratio of the stress difference *σ*_f2_–*σ*_f1_ to the corresponding strain difference, *ε*_f2_ (= 0.25%)–*ε*_f1_ (= 0.05%). It can be calculated using the equation:

(2) $$E_{f}=\frac{\sigma_{f2}-\sigma_{f1}}{\varepsilon_{f2}-\varepsilon_{f1}}$$

where *σ*_f1_ (MPa) is the flexural stress at deflection *S*_1_, *σ*_f2_ (MPa) is the flexural stress at deflection *S*_2_, and *ε*_f_ is the flexural strain (*ε*_f2_ = 0.0025, *ε*_f1_ = 0.0005). 

For the determination of the flexural modulus, deflections *S*_1_ and *S*_2_ corresponding to values of the flexural strain of *ε*_f1_ and *ε*_f1_ can be calculated using the equation:

(3) $$S_{i}=\frac{\varepsilon_{\mathrm{fi}}\cdot L^{2}}{6h}$$

where *S*_i_ (mm) is the deflection and *ε*_fi_ is the corresponding flexural strain.

Testing was performed on the Beta 50-5 tensile testing machines (Messphysik materials testing GMBH, Fürstenfeld, Austria). The specimen was set on two supports and loaded with force *F* acting on the specimen midway between the supports. The loading of the specimen was performed while controlling the displacement speed at 2 mm/min.

## 3. Results and Discussion

### 3.1. Characterization of Nanocomposite Structure

The obtained polymer structures were characterized by means of X-ray diffraction (XRD) and scanning electron microscopy (SEM). XRD analysis is one of the most common methods used for investigating the structure of polymeric nanocomposites [2,7]. Characterization was performed on specimens made according to experiment No. 1, 5, 8, and 9 as well as on specimens made of pure PA12. The low proportion of nanoparticles used and their sizes made the observation of the morphological and structural features very challenging.

**Figure 2:**
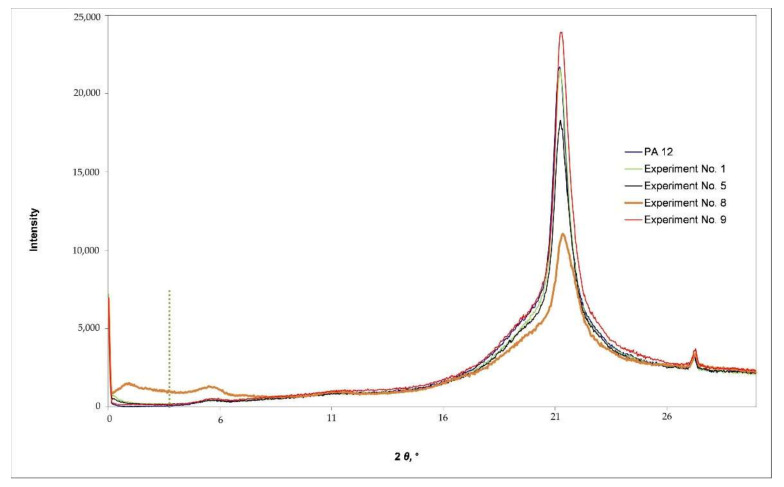
XRD diffractograms of PA12 and PA12/Cloisite 93A specimens.

The XRD diffractograms are given in Figure 2. The dotted vertical line in Figure 2 designates the value of the spacing between the layers of Cloisite 93A. According to the manufacturer’s declaration, the spacing was 2.36 nm, resulting in a diffraction angle (2*θ*) of 3.7°.

The XRD diffractogram in Figure 2 presents reflection peaks at 2*θ* = 6.1° and 2*θ* = 21.4°. These are typical for structures made of pure PA12. It is evident that there was no shift at all, suggesting that no crystal phase transformation or new crystal formation occurred when Cloisite 93A were introduced into the PA12 matrix [38,39]. A reflection peak at a lower value of diffraction angle was observed for the specimen made according to experiment No. 8 as a result of the increased spacing between layers of nanofiller (from 2.36 to 4.41 nm), which indicates the intercalation of polymer chains within the layers of nanoclay [24,38].

The XRD diffractograms for specimens made according to experiments No. 1, 5, and 9 (Figure 2) show the absence of basal reflection d_001_ and indicate the exfoliation of nanofiller in the polymer matrix [2,7,24]. However, since the absence of diffraction maximum could also indicate the agglomeration of nanofiller, the microstructure of the specimens was additionally evaluated using SEM microscopy.

**Figure 3:**
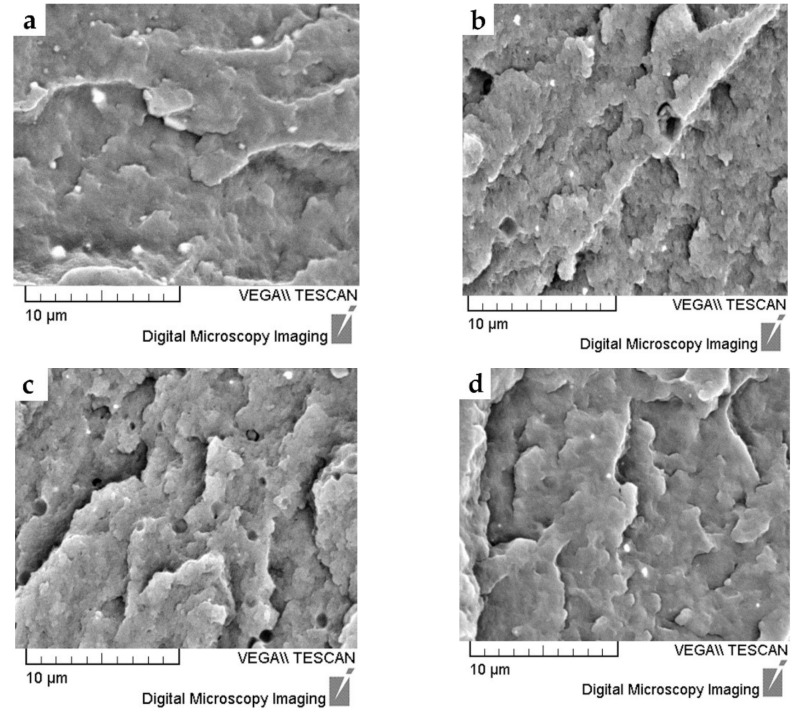
SEM micrographs of (**a**) specimens made according to experiment No. 1, (**b**) specimens made according to experiment No. 5, (**c**) specimens made according to experiment No. 8, and (**d**) specimens made according to experiment No. 9.

**Figure 4:**
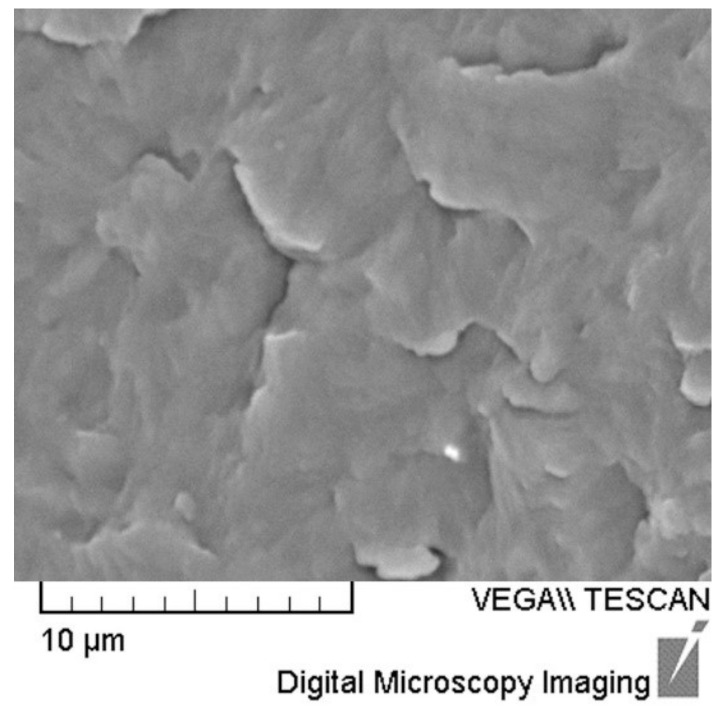
SEM micrograph of specimen made of pure PA12.

SEM micrographs of specimens made according to experiment No. 1, 5, 8, and 9 are given in Figure 3. Figure 4 shows the microstructure of specimen made out of pure PA12.

Thorough the visual investigation of a large number of SEM images of samples of polymer nanocomposites, it was revealed that the fillers could ne, in general, identified and characterized by the upper range of gray values (i.e., white or near white), while polymers are identified and characterized by the lower range of gray values (i.e., black or near black) [40].

The micrographs in Figure 3a,b,d, show the uniform dispersion of clay white nanoparticles, which is in accordance with the XRD diffractograms and the assumption of full exfoliation in these specimens. It could be concluded that a good level of exfoliation was reached. An exfoliated morphology is desired because of the large contact area between the polymer matrix and filler, resulting in optimal material properties [2,8,9]. 

The black color of nanoparticles in Figure 3c indicates the non-exfoliated layers of nanoclay in the PA12 matrix for Experiment No. 8. Larger black particles indicate nanofiller agglomeration, which is also in accordance with the XRD diffractogram of specimen 8. Figure 4 shows SEM micrographs of the specimen made out of pure PA12. 

It has to be mentioned that the complete exfoliation of clays in the polymer matrix is not easy to achieve because clay platelets tend to stack together into larger micron-sized aggregates held electrostatically with each other. This is especially true at high contents of nanoclay in PA12 matrix [2,7,8,9,38]. 

### 3.2. Flexural Properties of PA12/Clay Nanocomposites

**Figure 5:**
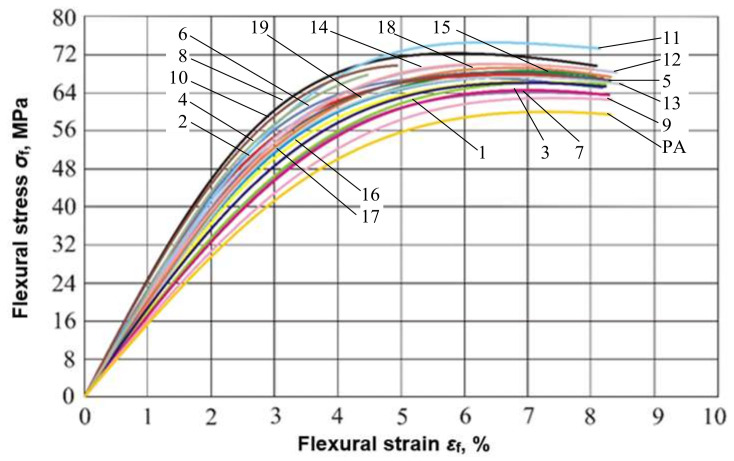
Flexural stress/strain plot for all experiments.

Curves of flexural stress versus flexural strain for all the experiments are given in Figure 5.

The values of flexural strength and flexural modulus for all the experiments are given in Table 3. These values are calculated using Equations (1) and (2), and represent the arithmetic mean of the results obtained for five test specimens of each experiment. 

#### 3.2.1. Flexural Strength

From Table 3, it can be observed that minimum and maximum response for flexural strength amounts to 62.2 and 72.2 MPa, respectively. The arithmetic mean of specimen responses is 67.9 MPa.

In order to estimate the suitable approximation between dependent and independent variables, four regression models (linear, two-factor interaction (2FI), quadratic, and cubic) were evaluated using the root mean square error, lack of fit, and R square metrics. Based on the results, the quadratic model was chosen as the most suitable for the estimation of the relationship between the flexural strength of the polymer nanocomposite and the three input process parameters (content of nanoclay, rotation frequency, and temperature). The analysis of variance was performed for the quadratic regression model and the results are given in Table 4. 

A *p*-value less than or equal to 0.05 is considered to be statistically significant. The *p*-value of the model (0.01) indicates that at least one of nine regression variables have a regression coefficient unequal to zero–i.e., they have a correlation with the dependent variable. The *p*-values for variable A and A^2^ are less than 0.05, meaning that they are statistically significant (have considerable effects on the response). Variables B, C, AB. AC, BC, B^2^, and C^2^ with *p*-values greater than 0.05 are not significant and could be excluded from the model. The lack of fit for the model is not significant (*p*-value = 0.65 is greater than 0.05) and implies that the proposed model fits the experimental data. The coefficient of determination is 0.83. 

All statistically insignificant variables were removed from the model using the backward-elimination rule and a reduced model was made. The analysis of variance was performed for the reduced model and the results are given in Table 5.

From Table 5, it can be seen that variables A and A^2^ are statistically significant (*p*-values are less than 0.05). The lack of fit for the model is not significant (*p*-value of 0.62 is greater than 0.05) and implies that the proposed model fits the experimental data. The coefficient of determination is 0.67. Based on the obtained results, an expression showing functional correlation between the flexural strength of PA12/clay nanocomposite and the content of nanoclay was established:

Flexural strength = 59.07 + 2.68· nanoclay content − 0.17· nanoclay content^2^ (4)

**Figure 6:**
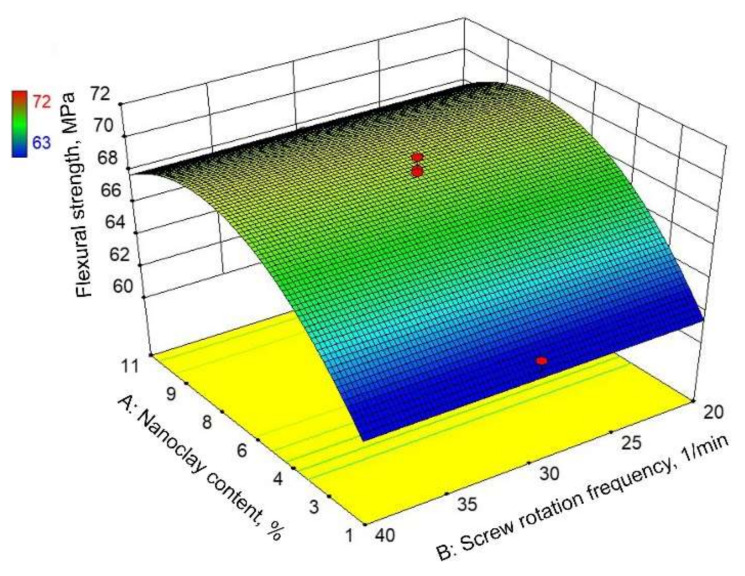
Response surface of reduced quadratic regression model: flexural strength.

**Figure 7:**
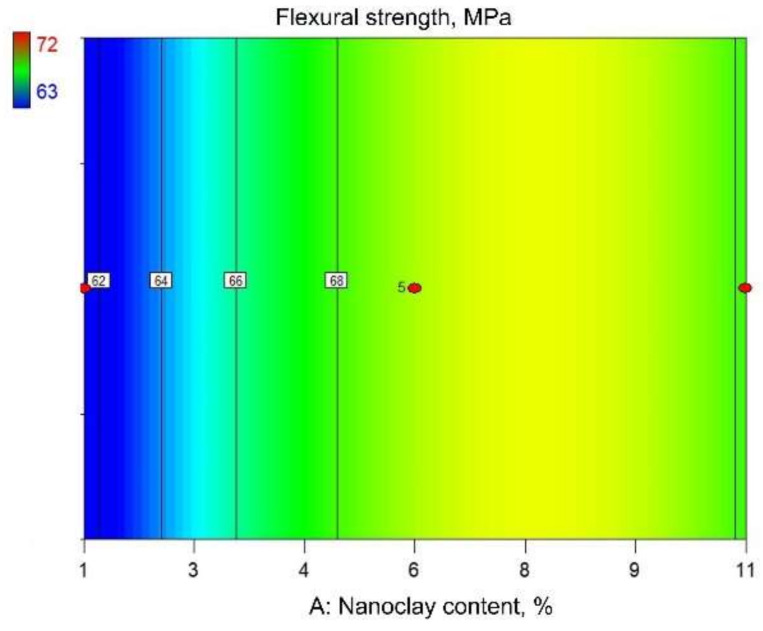
Contour view of reduced quadratic regression model: flexural strength.

Figure 6 and Figure 7 show graphical representations of the reduced quadratic regression model. The response surface presented in Figure 6 shows the estimated flexural strength dependent on the nanoclay content and the screw rotation frequency. According to Figure 6, nanoclay content has a significant influence on the flexural strength, where the maximum flexural strength value is attained at an approximately 8–8.5% nanoclay content. In contrast to the nanoclay content, the screw rotation frequency does not influence the flexural strength. This could also be concluded from the contour plot shown in Figure 7.

#### 3.2.2. Flexural Modulus

From Table 3, it can be observed that the minimum and maximum responses for flexural modulus amount to 1.4 and 2.2 GPa, respectively. The arithmetic mean of the specimen responses is 1.9 GPa.

In order to estimate a suitable approximation between the dependent and independent variables, four regression models (linear, two-factor interaction (2FI), quadratic, and cubic) were evaluated using the root mean square error, lack of fit, and R square metrics. Based on the results, the linear model was chosen as the most suitable for the estimation of the correlation between the flexural modulus of the polymer nanocomposite and three input process parameters (content of nanoclay, rotation frequency, and temperature). The analysis of variance was performed for the linear regression model and the results are given in Table 6.

The *p*-value of the model (**<** 0.0001) indicates that at least one of the three regression variables have a regression coefficient unequal to zero—i.e., they have a correlation with the dependent variable. The *p*-value for variable A is less than 0.05 and is statistically significant (has a considerable effect on the response). Variables B and C have *p*-values greater than 0.05, meaning that they are not significant and could be excluded from the model. The lack of fit for the model is not significant (*p*-value of 0.76 is greater than 0.05) and implies that the proposed model fits the experimental data. The coefficient of determination is 0.76. 

In the next step, all statistically insignificant variables were removed from the model using the backward-elimination rule and a reduced model was created. The analysis of variance was performed for the reduced model and the results are given in Table 7.

Table 7 shows that variable A is statistically significant (*p*-values are less than 0.05). The lack of fit for the model is not significant (p-value of 0.81 is greater than 0.05) and implies that the proposed model fits the experimental data. The coefficient of determination was 0.75. Based on the obtained results, an expression showing the functional correlation between the flexural modulus of PA12/clay nanocomposite and the content of nanoclay was established:

Flexural modulus = 1.4 + 0.07· nanoclay content(5)

**Figure 8:**
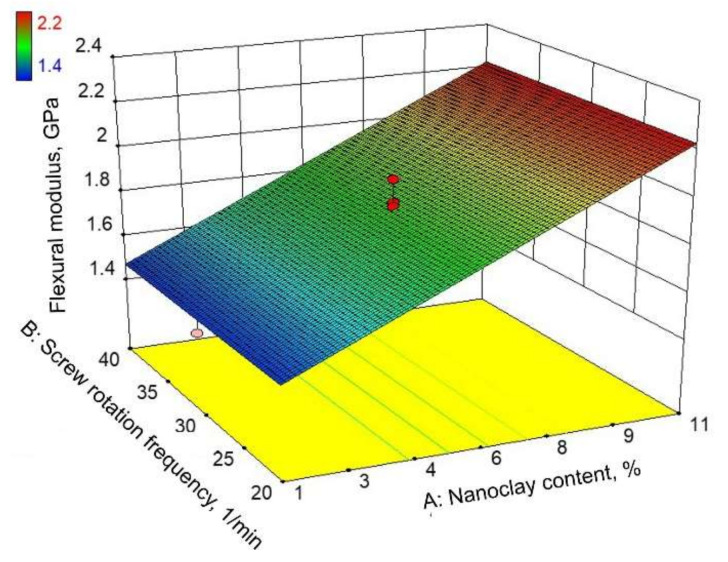
Response surface of reduced linear regression model: flexural modulus.

**Figure 9:**
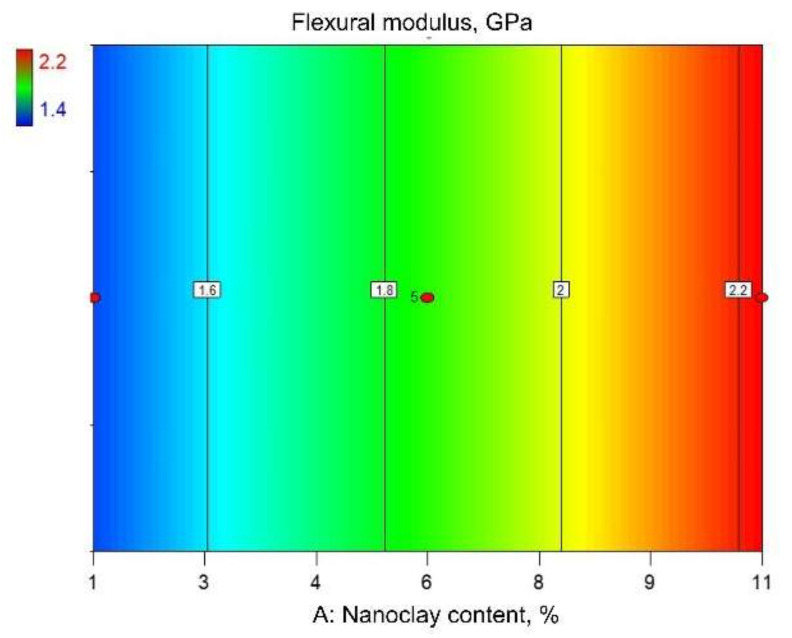
Contour view of reduced quadratic regression model: flexural strength.

Figure 8 and Figure 9 show graphical representations of the reduced linear regression model. Figure 8 represents the surface plot of Equation (5). It can be seen that the flexural modulus steadily increases with an increasing nanoclay content. As in the case of flexural strength, it can be observed that the screw rotation frequency does not affect the flexural modulus. All this can also be concluded from the contour plot shown in Figure 9.

## 4. Conclusions

In this study, the influence of the nanoclay content, screw rotation frequency, and mixing temperature on the flexural properties of PA12/clay nanocomposite was analyzed. The characterization of the microstructure by the means of XRD and SEM microscopy revealed the full exfoliation of nanofiller in specimens with a lower content of Cloisite 93 A (0.95% to 3%). 

Through structure characterization performed by X-ray diffraction (XRD) methods and scanning electronic microscopy (SEM), it was concluded that full exfoliation occurred in specimens with a lower Cloisite 93A content (0.95 to 3%). The more the nanoclay content increased, the less exfoliated and more intercalated the structure became.

Within statistical analysis, analyses of variance and regression analyses of the interdependence of flexural properties and mixing parameters were carried out, giving a thorough insight into how separate parameters influenced the observed properties. The analysis of variance showed that only the nanoclay content had a significant influence on the flexural properties, while the mixing temperature and screw rotation frequency had no influence on the observed properties (*p*-value greater than 0.05).

Through the use of regression analysis, expressions showing the correlation of significant mixing parameters with flexural strength and the flexural modulus of nanocomposites PA12/Cloisite 93A were determined. The obtained expression was valid for the mixing of PA12-based nano composites reinforced with Cloisite 93A on a Brabender extrusion line equipped with a twin-screw extruder, as used in this research, as well as for a range of the analyzed parameters’ values.

From the test results, it is apparent that an increase in nanoclay content from 1 to 8% significantly increases the flexural strength, while any further increase in the nanoclay content slightly decreases the flexural strength. An increase in nanoclay content also significantly increases the flexural modulus. If separate specimens of PA12/Cloisite 93A nanocomposite are compared to pure PA12, the nanocomposite has a 19% higher flexural strength and 56% higher flexural modulus than pure PA12.

## Figures and Tables

**Table 1:** Central composite design of experiment with physical values of mixing parameters.

Experiment No./Point of the Experiment	A: Nanoclay Content, %	B: Screw Rotation Frequency, min^−1^	C: Mixing Temperature, °C
1/factorial point	3	20	210
2/factorial point	9	20	210
3/factorial point	3	40	210
4/factorial point	9	40	210
5/factorial point	3	20	230
6/factorial point	9	20	230
7/factorial point	3	40	230
8/factorial point	9	40	230
9/axial point	0.95	30	220
10/axial point	11.05	30	220
11/axial point	6	13.18	220
12/axial point	6	46.82	220
13/axial point	6	30	203.18
14/axial point	6	30	236.82
15/center point	6	30	220
16/center point	6	30	220
17/center point	6	30	220
18/center point	6	30	220
19/center point	6	30	220

**Table 2:** Technical data of Brabender extrusion line [33,34,35].

Extruder type	Twin-screw, counter-rotating
Screw diameter D	42 mm
Length/diameter ratio L/D	6
Operating temperature max.	350 °C
Conveyor belt speed	0.6 to 6 m/min
Strand diameter	1 to 4 mm
Pellet length	3 mm
Strand pelletizer speed	0.5 to 15 m/min

**Table 3:** Test results of flexural strength and flexural modulus for all experiments.

Experiment No.	A: Nanoclay Content, %	B: Screw Rotation Frequency, min^−1^	C: Mixing Temperature, °C	Flexural Strength *σ*_fM_, MPa	Flexural Modulus *E*_f_, GPa
1	3	20	210	65.6	1.7
2	9	20	210	67.9	2.0
3	3	40	210	64.4	1.6
4	9	40	210	68.5	2.0
5	3	20	230	66.2	1.7
6	9	20	230	68.7	2.2
7	3	40	230	63.8	1.5
8	9	40	230	72.2	2.2
9	0.95	30	220	62.5	1.4
10	11.05	30	220	67.6	2.1
11	6	13.18	220	71.9	1.9
12	6	46.82	220	70.1	2.0
13	6	30	203.18	68.6	1.9
14	6	30	236.82	69.8	1.9
15	6	30	220	69.4	1.6
16	6	30	220	67.3	1.9
17	6	30	220	70.1	2.0
18	6	30	220	69.2	1.9
19	6	30	220	66.1	1.7
PA12				60.7	1.4

**Table 4:** Analysis of variance for the quadratic regression model: flexural strength.

Source	Sum of Squares	Degrees of Freedom df	Mean Square	*F* Value	*p*-Value
Model	101.68	9	11.30	4.85	0.01
A—Nanoclay content	48.82	1	48.82	20.94	0.001
B—Screw rotation frequency	0.50	1	0.50	0.21	0.66
C—Mixing temperature	3.11	1	3.11	1.34	0.28
AB	7.18	1	7.18	3.08	0.11
AC	2.62	1	2.62	1.12	0.32
BC	0.34	1	0.34	0.15	0.71
A^2^	28.79	1	28.79	12.35	0.01
B^2^	5.57	1	5.57	2.39	0.16
C^2^	0.0009	1	0.0009	0.0004	0.99
Residual	20.98	9	2.33		
Lack of fit	9.76	5	1.95	0.70	0.65
Pure error	11.21	4	2.80		
Cor total	122.7	18			

**Table 5:** Analysis of variance for the reduced quadratic regression model: flexural strength.

Source	Sum of Squares	Degrees of Freedom df	Mean Square	*F* Value	*p*-Value
Model	82.24	2	41.12	16.28	0.0001
A—Nanoclay content	48.82	1	48.82	19.33	0.001
A^2^	33.42	1	33.42	13.23	0.002
Residual	40.42	16	2.53		
Lack of fit	29.20	12	2.43	0.87	0.62
Pure error	11.21	4	2.80		
Cor total	122.66	18			

**Table 6:** Analysis of variance for the linear regression model: flexural modulus.

Source	Sum of Squares	Degrees of Freedom df	Mean Square	*F* Value	*p*-Value
Model	0.69	3	0.23	15.66	<0.0001
A—Nanoclay content	0.69	1	0.69	46.47	<0.0001
B—Screw rotation frequency	0.002	1	0.002	0.11	0.74
C—Mixing temperature	0.006	1	0.01	0.41	0.53
Residual	0.22	15	0.02		
Lack of fit	0.14	11	0.01	0.63	0.76
Pure error	0.08	4	0.02		
Cor total	0.92	18			

**Table 7:** Analysis of variance for the reduced linear regression model: flexural modulus.

Source	Sum of Squares	Degrees of Freedom df	Mean Square	*F* Value	*p*-Value
Model	0.69	1	0.69	50.90	<0.0001
A—Nanoclay content	0.69	1	0.69	50.90	<0.0001
Residual	0.23	17	0.01		
Lack of fit	0.15	13	0.01	0.56	0.81
Pure error	0.08	4	0.02		
Cor total	0.92	18			

## Data Availability

Not applicable.
